# Activin B is a novel biomarker for chronic fatigue syndrome/myalgic encephalomyelitis (CFS/ME) diagnosis: a cross sectional study

**DOI:** 10.1186/s12967-017-1161-4

**Published:** 2017-03-16

**Authors:** Brett A. Lidbury, Badia Kita, Donald P. Lewis, Susan Hayward, Helen Ludlow, Mark P. Hedger, David M. de Kretser

**Affiliations:** 1 Pattern Recognition and Pathology, Department of Genome Sciences, The John Curtin School of Medical Research, The Australian National University, Canberra, ACT 2601 Australia; 2 Paranta Biosciences Limited, Caulfield North, VIC 3161 Australia; 3 CFS Discovery, Donvale Medical Specialist Centre, Donvale, VIC 3111 Australia; 4 The Hudson Medical Research Institute, Monash University, Clayton, VIC 3168 Australia; 5 Centre for Proteins and Peptides, School of Life Sciences, Oxford Brookes University, Headington, Oxford, OX3 0BP UK; 6 Department of Anatomy and Developmental Biology, Monash University, Clayton, VIC 3168 Australia; 7 The National Centre for Epidemiology and Public Health, The Research School of Population Health, ANU, Canberra, ACT 2601 Australia

**Keywords:** Myalgic encephalomyelitis (ME), Chronic fatigue syndrome (CFS), Biomarker, Activins, Diagnosis

## Abstract

**Background:**

Investigations of activin family proteins as serum biomarkers for chronic fatigue syndrome/myalgic encephalomyelitis (CFS/ME). CFS/ME is a disease with complex, wide-ranging symptoms, featuring persistent fatigue of 6 months or longer, particularly post exertion. No definitive biomarkers are available.

**Methods:**

A cross-sectional, observational study of CFS/ME patients fulfilling the 2003 Canadian Consensus Criteria, in parallel with healthy non-fatigued controls, was conducted. Comparisons with a previously defined activin reference population were also performed. For the total study cohort the age range was 18–65 years with a female: male participant ratio of greater than 3:1. All participants were assessed via a primary care community clinic. Blood samples were collected for pathology testing after physical examination and orthostatic intolerance assessment. Cytokines, activin A, activin B and follistatin were also measured in sera from these samples. All data were compared between the CFS/ME and control cohorts, with the activins and follistatin also compared with previously defined reference intervals.

**Results:**

Serum activin B levels for CFS/ME participants were significantly elevated when compared to the study controls, as well as the established reference interval. Serum activin A and follistatin were within their normal ranges. All routine and special pathology markers were within the normal laboratory reference intervals for the total study cohort, with no significant differences detected between CFS/ME and control groups. Also, no significant differences were detected for IL-2, IL-4, IL-6, IL-10, IL-17A, TNF or IFN-gamma.

**Conclusion:**

Elevated activin B levels together with normal activin A levels identified patients with the diagnostic symptoms of CFS/ME, thus providing a novel serum based test. The activins have multiple physiological roles and capture the diverse array of symptoms experienced by CFS/ME patients.

## Background

Chronic fatigue syndrome/myalgic encephalomyelitis (CFS/ME) currently presents a diagnostic dilemma because of the multi-system nature of the symptoms and the lack of a definitive serum-based diagnostic. Diagnosis is currently based on clinical case definition criteria, of which the CDC-1994/Fukuda case definition is the most frequently applied [1], as evidenced from the twenty case definitions identified for systematic review (SR) by Brurberg et al. [2]. The same study also found few validation studies performed using the various diagnostic criteria, confirming the nebulous aspects of diagnosis by case definition.

Efforts to improve the rigour of CFS/ME case definitions have included an update of the 2003 Canadian Criteria to the International Consensus Criteria in 2011 [3, 4], which emphasises ME symptoms associated with cognitive and physical deficiency, with less importance on psychiatric co-morbidity and depression when compared to the Fukuda criteria. A more recent attempt to design clearer and simpler diagnostic criteria focused on post-exertional fatigue, and proposing the new name Systemic Exertion Intolerance Disease (SEID) [5], instead of ME and/or CFS. However, the diagnosis of the disease remains a challenge as the diagnostic criteria requires that patients experience the symptoms for at least 6 months before a definitive diagnosis can be made, leaving patients to struggle with the illness for a considerable time before being diagnosed.

Given that many patients often describe an influenza-like illness preceding the development of CFS/ME, the possibility that cytokines may provide diagnostic markers of CFS/ME has been assessed, but with little consensus as to their diagnostic utility, possibly influenced by disease-stage and other inter-patient variability. Evidence that the immune system is involved in CFS/ME is supported by low natural killer cell cytotoxicity [6], but to date an immune biomarker for CFS/ME has not been identified.

Given the accumulating data that activin A and B are involved in the control of inflammation and muscle mass [7], we undertook a cross-sectional study on the potential of activin A, activin B, and its binding protein (follistatin), as serum markers of CFS/ME, via a Melbourne clinic that works exclusively with CFS/ME patients. The activins are members of the Transforming Growth Factor β (TGFβ) family of proteins, and are dimers of subunits encoded by the *INHBA* (β_A_) and *INHBB* (β_B_) genes. The subunits dimerise to form activin A (β_A_β_A_) and activin B (β_B_β_B_) [7]. Our previous data show that in patients admitted to intensive care units (ICU) with acute respiratory distress, survival of up to 12-months post ICU can be predicted by the measurement of activin A and B levels during the first 5 days in ICU [8]. This may be a causal relationship, since increased activin A and activin B levels can induce a loss of muscle mass [9]. Interestingly, activin B is involved in inflammatory-induced anaemia via regulation of hepcidin expression [10], a function distinct from activin A.

In addition to immune dysregulation, the loss of muscle mass suggests that the activins are potentially involved in the pathogenesis of CFS/ME, given the prominence of muscle weakness and pain as diagnostic criteria across the various case definitions. Due to these associations, in addition to symptoms spanning cognitive and gastro-intestinal functions, it was hypothesised that significant fluctuations of serum activin/follistatin levels and ratios were likely for CFS/ME patients formally diagnosed under the 2003 Canadian Criteria [3]. Here we report the outcomes of a study of serum activin A, B and follistatin levels in blood samples from 45 patients diagnosed with CFS/ME under the 2003 Canadian Criteria, whose clinical findings formed a previously reported study and database [11].

## Methods

### Participants and setting

Participants in this study included 45 patients (40 females and 5 males: age 19–66 years) diagnosed with chronic fatigue syndrome at the CFS Discovery Clinic, a primary care community clinic that works exclusively with CFS/ME patients, located in Donvale (Victoria, Australia). Patients were diagnosed with CFS/ME if they fulfilled the Canadian Diagnostic Criteria, [3] as conducted by a medical practitioner and registered nurse both with 15–20 years’ experience with CFS/ME patients. For the CFS/ME cohort, length of illness ranged from 2 to 40 years. During the early phase of the study, the 2011 International Criteria for CFS/ME diagnosis were published, [4] but since the study had commenced participant recruitment and assessment under the 2003 criteria, the application of the 2003 criteria persisted for the duration of this study.

The study also recruited 17 healthy control participants, comprising 13 females and 4 males (age 24–60 years). Control participants were recruited via neighbouring clinics within the Doncaster district of East Melbourne, through CFS support groups and by word-of-mouth.

Research participants for this study were recruited over 2011–2012 from patients attending CFS Discovery for the first time, often after long periods of interaction with the medical profession, but no conclusive diagnosis or successful treatment. While there are theories, the cause and/or exposure that results in CFS/ME symptoms are not known. A cross-sectional (observation) study design was applied, with CFS/ME cases and healthy controls attending only once for research involvement over the 2011–2012 study period. Follow-up was provided as routine clinic attendance after the research interaction.

### Study details

The assessment of patients at CFS Discovery was previously reported by Reynolds et al. [11] which included the protocol for orthostatic intolerance testing and results for autonomic markers of cardiac and circulatory function in CFS/ME patients, with key pathology and cytokine markers for this cohort summarised in Table 1. In addition to the formal CFS/ME diagnostic criteria, assessed by survey and physical examination, research participants were also screened for potentially confounding comorbidities. The study contained tests and clinical assessments to exclude hypothyroidism, Lupus, Fibromyalgia (FM) and Multiple Chemical Sensitivity via the physical and mental status examinations. Also, conducted on each CFS/ME and control participant were routine blood and urine tests, as described below and Table 1. Assessments of sleep patterns were ascertained via the Epworth Scale, and the DASS-36 scale applied to assess anxiety, depression and generalised stress. Recommended guidelines for CFS research also were consulted for this investigation [12], allowing consistent comparisons to other studies. The “Minimal Requirements” under these guidelines were generally captured by the application of the Canadian Criteria to CFS/ME status.

**Table 1:** CFS/ME and healthy study control participant profile comparisons via representative pathology blood markers and cytokines

Assay or measurement	Study cohort	Mean ± SEM	*p* value
Age (years)	Control	36.83 ± 2.03	0.78
	CFS/ME	37.69 ± 1.78	
Haemoglobin (g/L)	Control	140.89 ± 2.48	0.64
	CFS/ME	139.13 ± 2.12	
Thyroid stimulating hormone (mIU/L)	Control	1.38 ± 0.16	0.72
	CFS/ME	1.45 ± 0.10	
Vitamin D (nmol/L)	Control	73.61 ± 7.73	0.71
	CFS/ME	76.64 ± 3.99	
Anion gap (mmol/L)	Control	11.49 ± 0.55	0.14
	CFS/ME	10.62 ± 0.30	
GTT–blood glucose 30 min post load (mmol/L)	Control	7.15 ± 0.52	0.45
	CFS/ME	7.66 ± 0.27	
GTT–blood insulin 30 min post load (mU/L)	Control	50.88 ± 9.80	0.07
	CFS/ME	81.33 ± 6.76	
Interleukin-2 (pg/mL)	Control	0.22 ± 0.12	0.09
	CFS/ME	0.54 ± 0.11	
Interleukin-4 (pg/mL)	Control	0.26 ± 0.14	0.15
	CFS/ME	0.55 ± 0.11	
Interleukin-6 (pg/mL)	Control	0.86 ± 0.25	0.20
	CFS/ME	1.37 ± 0.20	
Interleukin-10 (pg/mL)	Control	1.06 ± 0.16	0.43
	CFS/ME	1.18 ± 0.09	
Interleukin-17A (pg/mL)	Control	Not detected	N/A
	CFS/ME	Not detected	
Interferon-γ (pg/mL)	Control	0.57 ± 0.20	0.31
	CFS/ME	0.37 ± 0.11	
Tumour necrosis factor (pg/mL)	Control	Not detected	N/A
	CFS/ME	Not detected	

Analyses comprised healthy controls (n = 17) and the CFS/ME cohort (n = 45), except for cytokine and GTT studies that compared n = 8–10 controls with n = 38–44 CFS/ME. Statistical significance was calculated by unpaired Mann–Whitney test

*GTT* glucose tolerance test

A patient was classified as having CFS/ME if she or he met the above criteria and all other possibilities were excluded as listed above. If a patient’s fatigue was not severe enough, or if the symptom criteria for CFS/ME were not met, he or she was classified as having idiopathic chronic fatigue. Seventeen healthy controls were also recruited and assessed exactly as described for CFS/ME participants, including orthostatic intolerance testing to screen for autonomic comorbidities. Healthy participants could not be a relative of a previous or current CFS/ME patient, or live at the same residential address.

All research participants were non-identifiable to the researchers, with individual identifiers removed from the data provided, except for a unique identification (ID) code. Because only ID codes were provided, no clinical details were attached to samples for activin/follistatin, cytokines and pathology testing, removing potential bias from test result reporting.

As a first attempt at a cross-sectional study on this CFS/ME cohort, a minimum of 30 participants for each group was planned for statistical robustness, and was achieved for the CFS/ME group. Only 17 healthy control participants were recruited within the designated study period, in spite of proactive study advertising and connection with other GP clinics in the district. In addition to the 17 recruited study controls, comparisons of serum activin/follistatin levels with a previously recruited healthy reference population (n = 141) [8] were also conducted for validation.

### Patient involvement

No patients were involved in the conception or design of this study, nor were patients asked for input at any stage of the project. A summary of project results is available to research patients, as stated in the participant information pack, on request.

### Activin and follistatin analyses

Non-fasting blood samples were collected from patients after the 20-min standing test and the concentrations of activin A, activin B and follistatin were measured in the serum isolated from these samples. Concentrations of activin A were determined using a two-site ELISA (using antibodies supplied by Oxford Brookes University) as previously published [13]. This assay measures both free and follistatin-bound activin A dimers and has no significant cross-reaction with other activin isoforms, such as activin B. Activin B was measured by two-site ELISA, as previously described (using antibodies supplied by Oxford Brookes University) [14]. Follistatin concentrations were determined using an extensively validated radioimmunoassay [15]. Mean activin/follistatin concentrations were compared between the CFS/ME and control cohorts recruited for this study. Further analyses were performed comparing the recruited CFS/ME cohort to data previously collected to calculate reference intervals for each marker.

### Pathology and cytokine analyses

In addition to the blood samples collected for activin/follistatin testing, blood was also drawn for pathology testing at Australian Clinical Laboratories (Clayton, Victoria). Tests performed on each CFS/ME and control participant were routine full (complete) blood count examinations, multiple biochemical analyses that included liver and kidney function tests (with 24-h urine analysis), vitamin D (25-OH), parathyroid hormone (PTH), dehydroepiandrosterone sulfate (DHEAS), immunoglobulin (Ig) E, antinuclear antibodies (ANA) and thyroid stimulating hormone (TSH). A fasting glucose tolerance test (GTT) was performed on a separate day post clinic consultation with blood glucose and insulin measured before glucose load (75 g in 200 mL), then at 30 and 60 min post glucose load.

Chronic fatigue syndrome/myalgic encephalomyelitis and control sera were also investigated for interleukin-2 (IL-2), interleukin-4 (IL-4), interleukin-6 (IL-6), interleukin-10 (IL-10), interleukin-17A (IL-17A), tumour necrosis factor (TNF) and interferon-gamma (IFN-γ). The seven cytokines were simultaneously quantitated via multiplex cytometric bead array (human CBA kit, catalogue number 560484, BD Biosciences, North Ryde, NSW), with data acquisition and analysis conducted on a BD FACS Calibur (JCSMR FACS Unit, ANU, Canberra).

### Statistical analyses

Comparisons of means or medians between CFS/ME and control groups were performed by independent t test or Mann–Whitney tests respectively, depending on data distribution within the sample and whether the data were continuous or ordinal, with statistical significance accepted at p < 0.05. Whether data followed a normal distribution, or not, was determined by a one-sample Kolmogorov–Smirnov (K–S) Test (SPSS version 22). Data are presented as mean ± SEM.

Chi square (χ^2^) was calculated to determine the statistical significance (p < 0.05) of the frequency of elevated anti-nuclear antibodies (ANA ≥ 160) between CFS/ME and study control cohorts. Receiver operating curves (ROC) were constructed using SPSS (version 22.0) software, and calculated the area under curve (AUC ± SEM) to distinguish CFS/ME from healthy control cases, using a non-parametric model.

Pathology and activin/follistatin results had no missing data. Missing data was common for the seven serum cytokines measured, and recorded as not detected (ND). Cases with missing data were excluded from the analyses. Mean ± SEM were not reported for TNF and IL-17A since protein was detected in only 2–3 samples across the CFS/ME and control cohorts.

### Study approval

All research participants included in this study provided full, signed consent as dictated by the guidelines of the ANU Human Research Ethics Committee (ANU-HREC). This study and associated protocols were conducted after approval by the ANU-HREC (HREC identification 2011/031).

## Results

### Features of CFS/ME cohort

Routine medical and pathology analyses of 45 patients (40 females and 5 males, age 19–66 years, length of illness 2 to 40 years) diagnosed with CFS/ME under the Canadian Diagnostic Criteria, and 17 healthy controls (13 females, 4 males; age 24–60 years), were conducted. Key assay markers measured in patients diagnosed with CFS/ME were all within laboratory reference intervals (Table 1). Other routine tests included liver function tests, urea, electrolytes and creatinine, as well as erythrocyte and leukocyte markers/indices, with all results falling within the laboratory reference interval for CFS/ME and control cohorts. In addition, dihydroepiandrosteronesulphate (DHEAS), parathyroid hormone (PTH) and IgE were measured, but also showed no significant differences (p = 0.39 to 0.83, Mann–Whitney). The frequency of positive antinuclear antibody (ANA) detection (≥160), a marker for autoimmune disorders, was not statistically significant (χ^2^ = 2.9, df = 1, p = 0.41) between CFS/ME and control groups. Also, no significant differences between CFS/ME and healthy participants were detected for blood glucose and insulin at 30-min after glucose loading, during an oral glucose tolerance test (GTT).

Table 2 summarises the physical characteristics and symptom profile against the Canadian Criteria for the CFS/ME cohort investigated. Healthy controls were significantly taller (p = 0.002), while diastolic blood pressure and pulse rate (p < 0.05) were significantly elevated for the CFS/ME cohort. All CFS/ME participants reported the key symptoms of fatigue for greater than 6 months and post-exertional fatigue (“payback”), as well as limited activity, unrefreshing sleep, impaired thinking/speech and orthostatic intolerance (Table 2). The prevalence of other symptoms under the Canadian Criteria varied from approximately 50% of the CFS/ME cohort to less than 10% (Table 2). Participants from the healthy control cohort occasionally reported headache, gut upset or difficulties waking from sleep, but these cases were sporadic and when reported involved only 1–2 participants from the total cohort.

**Table 2:** Summary of physical features and diagnostic symptom profile for the CFS/ME cohort investigated for cytokine and activin/follistatin variation

Measurement	Median (range)	
	CFS/ME cohort	Healthy control cohort
Height (cm)	165.0 (122–188)	171.5 (153–187)
Weight (kg)	60.0 (52–124)	66.0 (51–98)
BMI	22.2 (18.9–47.2)	21.9 (19.6–32.1)
Blood pressure		
Systolic (mmHg)	120.0 (102–168)	121.5 (107–140)
Diastolic (mmHg)	80.0 (65–104)	74.0 (55–90)
Pulse rate (bpm)	72.0 (51–102)	60.0 (43–89)
Epworth sleep scale	6.0 (0–20)	4.0 (0–15)

Canadian criteria symptoms (percentage)	CFS/ME cohort	
Reported by 100% of study participants	Fatigue ≥6 months	Post-exertional fatigue
	Activity limited	Stress with activity
	Unrefreshing sleep	Reduced concentration
	Impaired thinking + speech	Orthostatic intolerance
	Vertigo	Food intolerance
Reported by ~50% of study participants	Gut axis (constipation, indigestion, diarrhoea)	Sleep (difficulty to wake, sleeping during day)
	New allergies	Sighing breaths
	Arrhythmia	Confusion
		Smell sensitivity
Reported by ≤10% of study participants	Migraine headache	Sensitivity to touch
	High heart rate upright	Pain frequency

Epworth sleep scale: 0–10 (normal daytime sleepiness): 11–15 (excessive daytime sleepiness): 16–24 (severe daytime sleepiness)

Height was significantly greater for the healthy control cohort (p = 0.002, Mann–Whitney U). Diastolic blood pressure and pulse rate were significantly elevated for CFS/ME (p < 0.05, Mann–Whitney U). CFS/ME (n = 40 to 43); Healthy Controls (n = 7 to 17)

*BMI* body mass index, *bpm* beats per minute

### Activin and follistatin responses in CFS/ME and healthy control participants

**Fig. 1:**
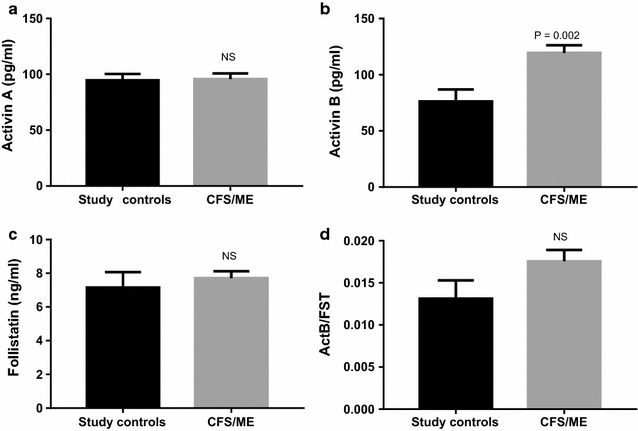
Mean serum concentrations of **a** activin A, **b** activin B and **c** follistatin levels in study participants diagnosed with chronic fatigue syndrome (CFS/ME), compared to a study control group comprising healthy participants (study controls). **d** Ratio of activin B to follistatin levels (ActB:FST). Data are presented as mean ± SEM

Measurement of activin A, activin B and follistatin levels in serum samples from the patient and control groups were conducted using validated assays to determine the clinical utility of these proteins as biomarkers. Levels of activin B, but not activin A and follistatin, were found to be significantly elevated (p = 0.002) in patient serum samples compared to control subjects (Fig. 1). The activin B response was unique as in addition to activin A, the levels of the other cytokines measured, namely IL-2, IL-4, IL-6, IL-10, IL-17A, TNFα and IFN-γ, were not statistically significant between groups (Table 1: IL-17A was detected in 2/45 CFS/ME samples and no controls, while serum TNFα was detected in 3/45 CFS/ME samples and no controls). Despite the significant elevation of activin B, the ratio of activin B to follistatin (ActB:FST), was not found to reach significance with respect to controls (p = 0.096) (Fig. 1d).

**Fig. 2:**
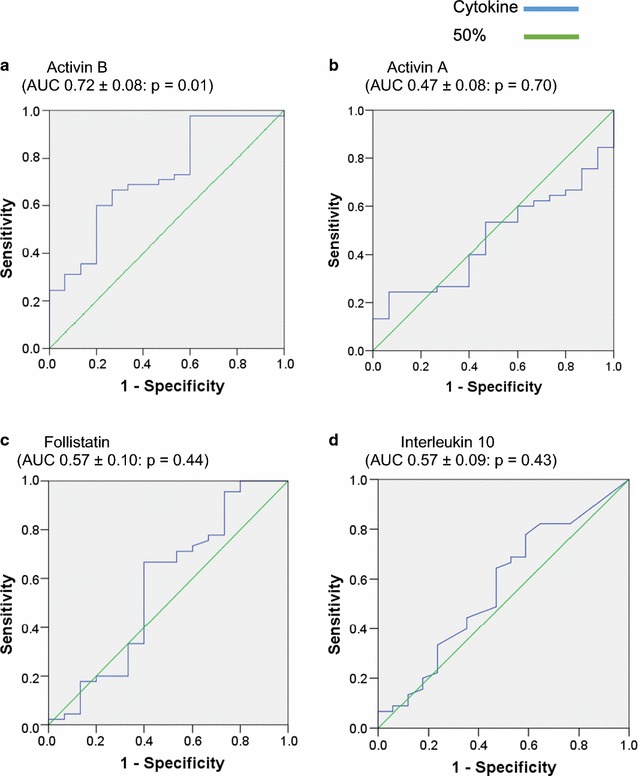
Receiver Operating Curves (ROC) for **a** activin B, **b** activin A, **c** follistatin and **d** interleukin 10 (IL-10), for separating CFS/ME participants from healthy study controls recruited at the same time. The *blue line* represents the cytokine and the *green line* the 50% threshold of Sensitivity versus 1–Specificity for the protein detected in the CFS/ME or control serum

Receiver operating curves (ROC) were calculated from the CFS/ME versus healthy study controls data for activin A, activin B, follistatin and IL-10. IL-10 was chosen since previous observations suggested a significant role for this cytokine in CFS/ME. The calculated AUC for activin B was 72.4% (0.724 ± 0.076), which was significantly independent (p = 0.010) from the 50% (0.5) AUC threshold (Fig. 2a). For activin A, follistatin and IL-10 (Fig. 2b–d), AUC results ranged from 0.467 to 0.567 and were not significant (p = 0.43–0.70).

### CFS/ME activin analyses in relation to a normal reference population

**Fig. 3:**
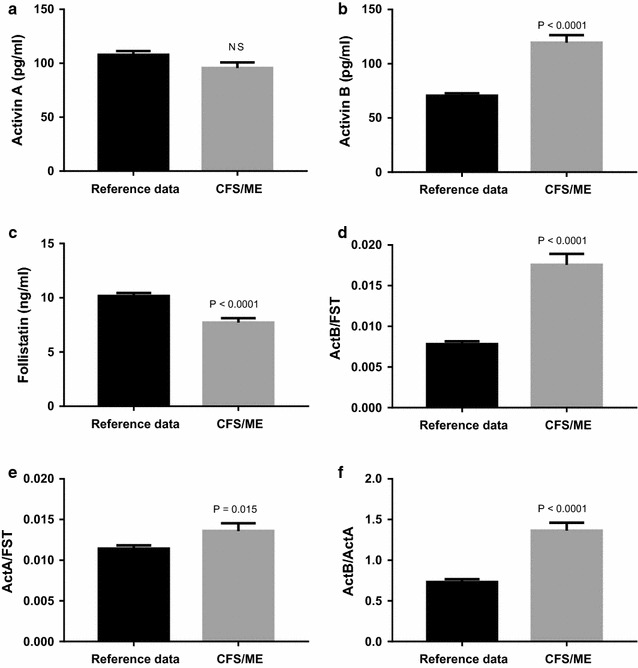
Mean serum concentrations of **a** activin A, **b** activin B and **c** follistatin levels in study participants diagnosed with chronic fatigue syndrome (CFS/ME) compared to activin/follistatin data from a reference population. Ratio of **d** activin B to follistatin levels (ActB:FST), **e** activin A to follistatin levels (ActA:FST) and **f** Ratio of activin B to activin A (ActB:ActA). Data are presented as mean ± SEM

Prior to this study, 141 healthy volunteers were recruited by de Kretser et al. [8] to determine the serum reference intervals for activin A, activin B and follistatin, including the ratios of the activins A and B to follistatin (ActA:FST and ActB:FST) in a normal population (referred to herein as reference data to distinguish it from the study control group). This reference data was established using the same validated assays used to measure the samples obtained for this study. When comparing the data to this reference range, CFS/ME patients had a significantly higher activin B (p < 0.0001), but not activin A levels (Fig. 3a, b), suggesting an important relationship between activin B and CFS/ME. The control group data were within the reference range.

Comparisons to the reference data also showed that follistatin levels were significantly lower in the CFS/ME group (p < 0.0001) (Fig. 3c), and therefore, both activin A:follistatin (ActA:FST, p < 0.015) and activin B:follistatin (ActB:FST, p < 0.0001) ratios were significantly higher in CFS/ME patients (Fig. 3d, e), with a greater difference seen for the activin B/follistatin ratio. Concordantly, activin B levels relative to activin A levels (ActB: ActA ratio) were significantly elevated in CFS/ME patients (p < 0.0001) compared to the reference data (Fig. 3f). These data show that activin B levels, as well as activin A or B to follistatin ratios, were elevated above the normal range in patients with CFS/ME.

## Discussion

Chronic fatigue syndrome/myalgic encephalomyelitis is a disorder that causes unexplained, persistent and sometimes disabling fatigue, with no definitive diagnostic tests available. Patients with this illness experience frustration due to health care provider difficulties in reaching a diagnosis of CFS/ME, compounded by the range and variation of the available diagnostic criteria [2]. It is estimated that around 0.4% of people world-wide suffer from CFS/ME [16] and the Centres for Disease Control and Prevention in the USA has declared it a priority disease, along with TB and AIDS. With the majority of affected patients not receiving the correct diagnosis, and therefore never receiving the proper medical care for their illness [5], there is a pressing need for a diagnostic biomarker to assist these patients.

The results of this study establish that patients with serum activin B levels significantly elevated above the established reference range associate with the symptoms of CFS/ME, in contrast to serum activin A. Our data, therefore, indicate that the pattern of elevated serum activin B together with normal activin A identified a group of patients with CFS/ME, as defined by the 2003 Canadian Criteria. Consequently, this pattern of serum activin A and B represents a diagnostic marker, in contrast to the failure of other cytokines to define patients with CFS/ME. This observation was further confirmed by the finding that serum activin A and B levels in the study control group, recruited at the same time as the CFS/ME participants, were not statistically different from the established reference ranges [8]. The altered patterns of activins contrast with the numerous studies of a wide range of serum markers that have been measured previously seeking a diagnostic test for CFS/ME [2–6]. Serum and blood markers available via pathology laboratories generally fall within the assay reference interval for CFS/ME patients, as observed for the participants of this study.

The repeat of this study with larger patient and control cohorts will be a priority for the future, as will the investigation of activin/follistatin in relation to other forms of fatigue. Bias was minimised through the coding regime used to protect research participant confidentiality. No clinical notes or personal details were attached to the activin samples at any time, and the researchers had no contact with patients directly or with their medical histories during the planning of the study, and interrogation of the data.

Prior studies on the value of cytokines as biomarkers is divided, with some studies promoting the efficacy of cytokine profiles when considering onset characteristics and longitudinal studies [17, 18]. In contrast, reviews of the literature concluded that CFS/ME does not involve immune dysfunction [19], a conclusion supported by experimental studies that included results from CFS/ME patients in the context of exercise and sleep deprivation [20]. It was noted that interleukin 10 (IL-10) provided diagnostic value, but required spinal fluid samples from CFS/ME patients [21].

Transforming growth factor-beta (TGF-β) likewise has been mentioned as a significant marker [22, 23]. TGF-β modulation is also of note because of its role in NK-cell regulation and suppression [24], with NK-cell dysfunction a consistent CFS/ME observation for 25 years [25]. TGF-β1 inhibited NK cell DNA synthesis and production of cytokines, as well as weakly inhibited cytotoxic activity [26], with the inhibition of NK cell activity by TGF-β later found to involve the inhibition of mTOR kinase signalling, including post IL-15 stimulation [27].

However, TGF-β1 has a history of challenges in terms of biomarker development, involving the need for special blood collection methods and the removal of platelets prior to assay, as well as early reports of up to tenfold variation in disease studies, in comparison to control subjects [28–30]. TGF-β1 variation due to exogenous and endogenous factors, therefore, limited its diagnostic reliability. This is not true for activin B. Activin B levels do not change with respect to age, sex, BMI, ethnicity, smoking, allergies or type of medication [8] and to date, activin B levels have not been reported to be elevated or reduced in other diseases. Together with an established reference interval [8, 14], activin B has the potential to be a biomarker of clinical utility for CFS/ME, once validated on additional cohorts and other clinical criteria [2].

Activin A is a known modulator of the inflammatory cascade with its role as a pro-inflammatory cytokine discovered following its rapid elevation after lipopolysaccharide (LPS) was administered to mice [31], and there is evidence that activin B exerts actions similar to activin A, for example in muscle wasting and renal injury [32, 33]. However, others have shown that activin B exerts distinct functions to activin A, such as regulating hepcidin expression during the pathogenesis of inflammation-induced anaemia via Smad 1/5/8 signalling, while activin A exerts its actions through Smad 2/3 signalling [10]. Our data shows that levels of activin A and inflammatory cytokines were not elevated in CFS/ME patients, indicating an absence of underlying inflammation in these patients. The observed elevation of activin B levels, as well as elevated activin to follistatin ratios, indicates an increased systemic bioavailability of activins; however, the mechanisms by which activin B is causally linked requires further investigation.

The finding of an elevated serum activin B level also raises the possibility that treatment with follistatin may be of value in the management of patients with CFS/ME, since follistatin, in addition to blocking the actions of activin A, also can block the actions of activin B. Further, treatment with follistatin has the potential advantage that in addition to attenuating the actions of the activins, it can also block the actions of myostatin, thus enhancing muscle mass [34].

## Conclusions

Given the inability to clearly diagnose CFS/ME by the measurement of other cytokines, the demonstration that the measurement of activins A/B and follistatin enabled the identification of a population of CFS/ME patients, diagnosed via the Canadian criteria, is significant. These results propose that measurements of the activins and follistatin will be of value in separating patients with CFS/ME from other disorders that also cause fatigue. These data strongly suggest the need for a larger prospective study of CFS/ME patients to determine whether the detection of elevated serum activin B and normal activin A levels can predict the subsequent course for such patients.

## Abbreviations

CFS: chronic fatigue syndrome
ME: myalgic encephalomyelitis
SR: systematic review
SEID: Systemic Exertion Intolerance Disease
ICU: intensive care unit
FM: fibromyalgia
GP: general practitioner
ELISA: enzyme-linked immune-sorbent assay
PTH: parathyroid hormone
DHEAS: dehydroepiandrosterone sulfate
Ig: immunoglobulin
ANA: antinuclear antibodies
TSH: thyroid stimulating hormone
GTT: glucose tolerance test
IL: interleukin
TNF: tumour necrosis factor
IFN: interferon
K-S: Kolmogorov-Smirnov (test)
SEM: standard error of the mean
χ^2^: Chi square
ROC: receiver operating curve
AUC: area under curve
ND: not detected
FST: follistatin
ActA:ActB: activinA:activinB
TGF-β: transforming growth factor-beta
NK: natural killer (cells)
mTOR: mammalian target of rapamycin
